# Relationship between Apparent Diffusion Coefficient and Tumour Cellularity in Lung Cancer

**DOI:** 10.1371/journal.pone.0099865

**Published:** 2014-06-11

**Authors:** Lihua Chen, Jiuquan Zhang, Yongfeng Chen, Wenwei Wang, Xiangdong Zhou, Xiaochu Yan, Jian Wang

**Affiliations:** 1 Department of Radiology, Southwest Hospital, Third Military Medical University, Chongqing, China; 2 Department of Radiology, PLA 101st Hospital, Wuxi Jiangsu, China; 3 Department of Respiratory Medicine, Southwest Hospital, Third Military Medical University, Chongqing, China; 4 Department of Pathology, Southwest Hospital, Third Military Medical University, Chongqing, China; National Cancer Institute, National Institutes of Health, United States of America

## Abstract

**Background and objective:**

To prospectively investigate the relationship between the apparent diffusion coefficient (ADC) and cellularity in lung cancer.

**Methods:**

Sixty patients histopathologically confirmed with lung cancer (41 men, 19 women) underwent diffusion-weighted magnetic resonance imaging of the chest (with *b values* of 50 and 1000 s/mm^2^). The median mean ADC (ADCmean) value and median minimum ADC (ADCmin) value within each primary tumour were calculated and compared with the median nucleo-cytoplasmic ratio (NCR), which was selected to represent the cellularity. The correlation between the NCR and ADCmean/ADCmin was calculated with SPSS 18.0 software.

**Results:**

The mean ADCmean values, ADCmin values and median NCR were (1.07±0.12)×10^−3^ mm^2^/s, (0.86±0.14)×10^−3^ mm^2^/s, and (14.9±2.6) %, respectively, in adenocarcinoma; (0.88±0.10)×10^−3^ mm^2^/s, (0.73±0.12)×10^−3^ mm^2^/s, and (20.6±4.4) %, respectively, in squamous cell carcinoma; and (0.89±0.13)×10^−3^ mm^2^/s, (0.67±0.13)×10^−3^ mm^2^/s, and (18.3±3.5) %, respectively in small cell lung cancer. The NCR of squamous cell carcinoma and small cell lung cancer is greater than that of adenocarcinoma (P<0.01 and P = 0.002, respectively). There was an inverse relationship between ADCmean/NCR and ADCmin/NCR (r = −0.60, P = 0.001 and r = −0.47, P<0.001, respectively).

**Conclusion:**

There is a significant inverse relationship between tumour cellularity and ADC in lung cancer. However, tumour cellularity most likely is not the sole determinant of the ADC.

## Introduction

Lung cancer comprises almost 25% of all cancer deaths worldwide, and its incidence rates have risen dramatically over the last few years [1]. Timely and accurate detection and assessment of tumour stage in lung cancer plays a crucial role in planning the appropriate therapy and determining the prognosis.

Diffusion-weighted imaging (DWI), which tracks the microscopic rate of water diffusion within tissues, is a new means of monitoring tumour progression and response to treatment. Because it provides information about tissue cellularity and the integrity of cell membranes, DWI has benefits over traditional anatomical MRI techniques [2]. With the advent of the parallel imaging technique and echo-planar MR imaging techniques, DW imaging of the abdomen and thoracic cavity has become possible with fast imaging times, which minimises the effects of gross physiologic motion from respiration and cardiac movement [2]. Different tumour tissues have different cellular structures, which lead to different ADC values. Previous studies conducted *in vitro* [3], [4] and in animal models [5], [6] showed that the ADC value is highly inversely correlated with tumour cellularity and could be used to predict the tumour grade and response to therapy. Recently, a systematic review and meta-analysis [7] based on currently available evidence also supports this view in patients. Traditionally, the application of DWI for evaluation of the chest has been limited by respiratory and cardiac motion, which cause severe motion artefacts. With the development of multichannel magnetic resonance equipment, and fast MR imaging techniques in combination with parallel images in the last ten years, DWI integrated with chest MRI has increasing applications in clinical practice [2], [8]. It has been reported that DWI is capable of effectively evaluating therapeutic efficacy after treatment [9]. Even more, it also can distinguish lymph nodes with metastatic lung cancer from those without [10], [11], central lung cancer from its associated atelectasis [12], and malignant solitary pulmonary nodules from benign ones [13]. However, until now, there have been few studies that have discussed whether this is true in patients with lung cancer [2].

Thus, the purpose of our study was to prospectively assess whether there is a relationship between the ADC and the histopathological tumour cellularity in lung cancer.

## Materials and Methods

### Patients

The Medical Research Ethics Committee of the Third Military Medical University (Chongqing, China) reviewed and approved the present study. Written informed consent was obtained from each participant prior to the study. Between February 2012 and April 2013, 79 consecutive patients suspected of having lung cancer were assessed for eligibility if the lesions in size were >20 mm. The size of the lesions was measured on CT. Sixty patients (41 males, 19 females) histopathologically confirmed with lung cancer were included (Figure S1). The primary tumour diameters were 2.4 ∼ 13.7 cm, mean (4.9±0.9) cm.

### MR Imaging and DWI Metrics Measurement

**Figure 1:**
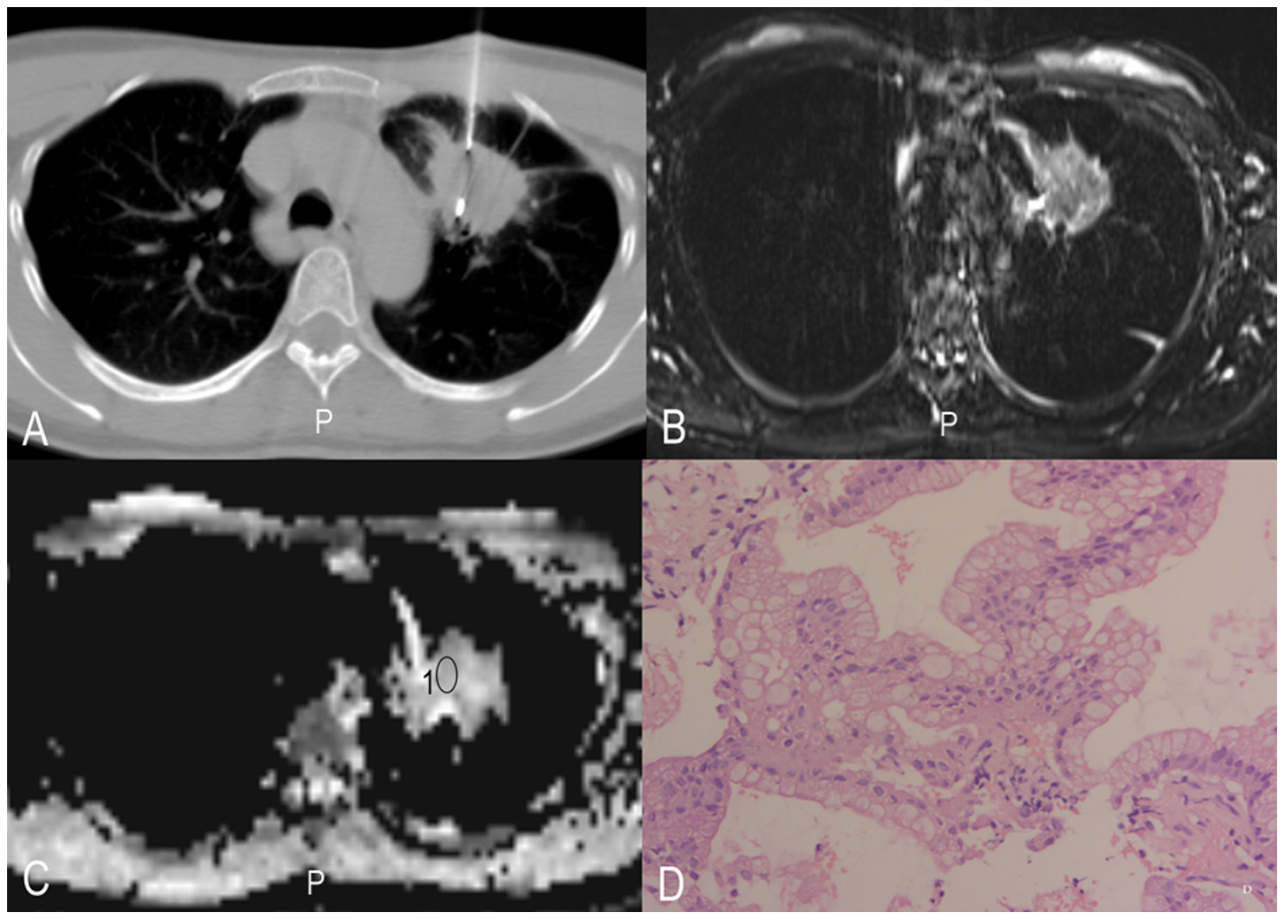
Histopathologic correlation of MR imaging data with biopsy specimens in a 59-year-old woman with adenocarcinoma. A, CT-guided biopsy; B, T2WI-TIRM; C, ADC map and ROI where ADCs have been measured are illustrated, 1 = ROI; D, corresponding histopathologic results for the tumour sample (original magnification, HE×400).

**Figure 2:**
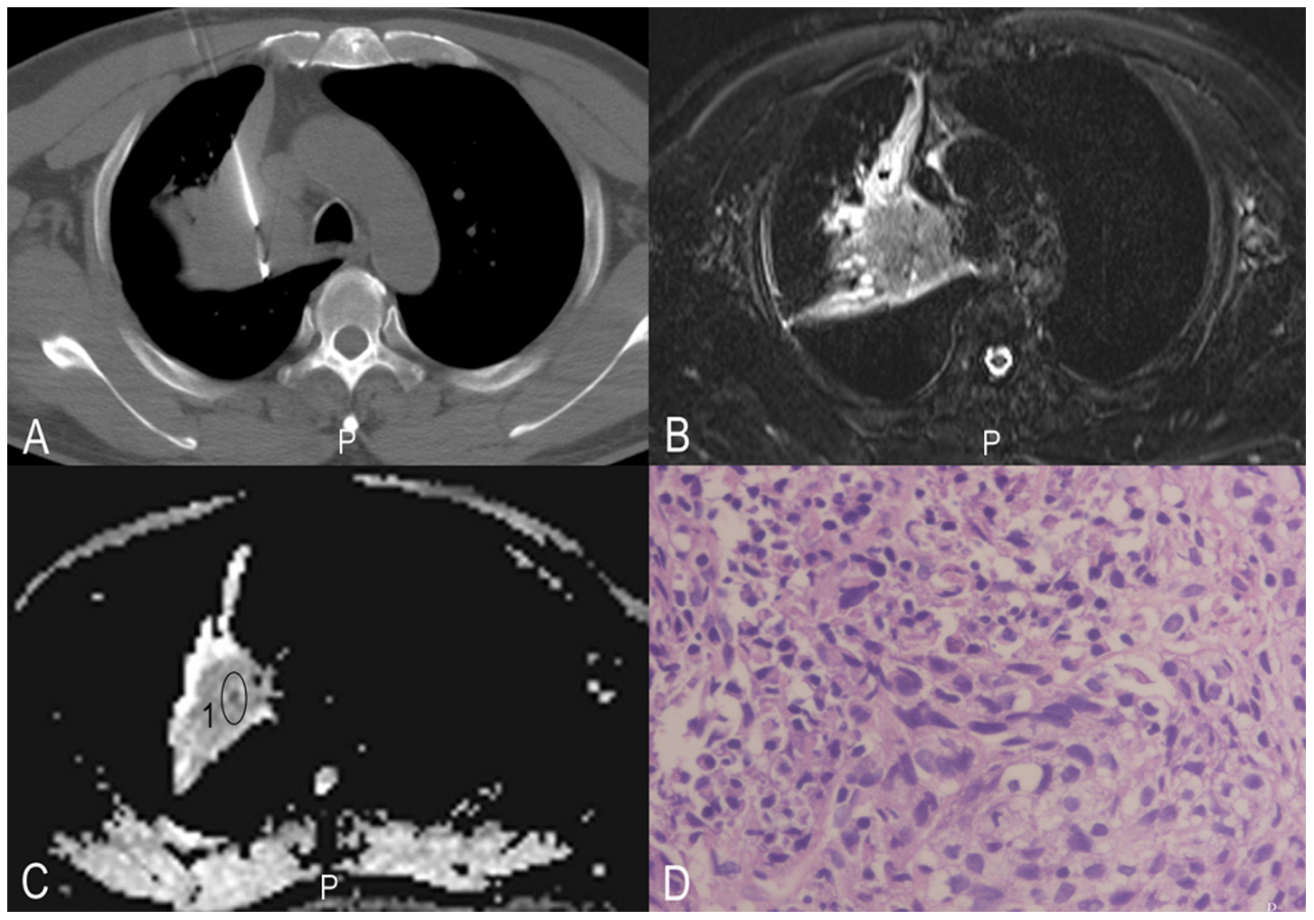
Histopathologic correlation of MR imaging data with biopsy specimens in a 33-year-old man with squamous cell carcinoma. A, CT-guided biopsy; B, T2WI-TIRM; C, ADC map and ROI where ADCs have been measured are illustrated, 1 = ROI; D, corresponding histopathologic results for the tumour sample (original magnification, HE×400).

**Figure 3:**
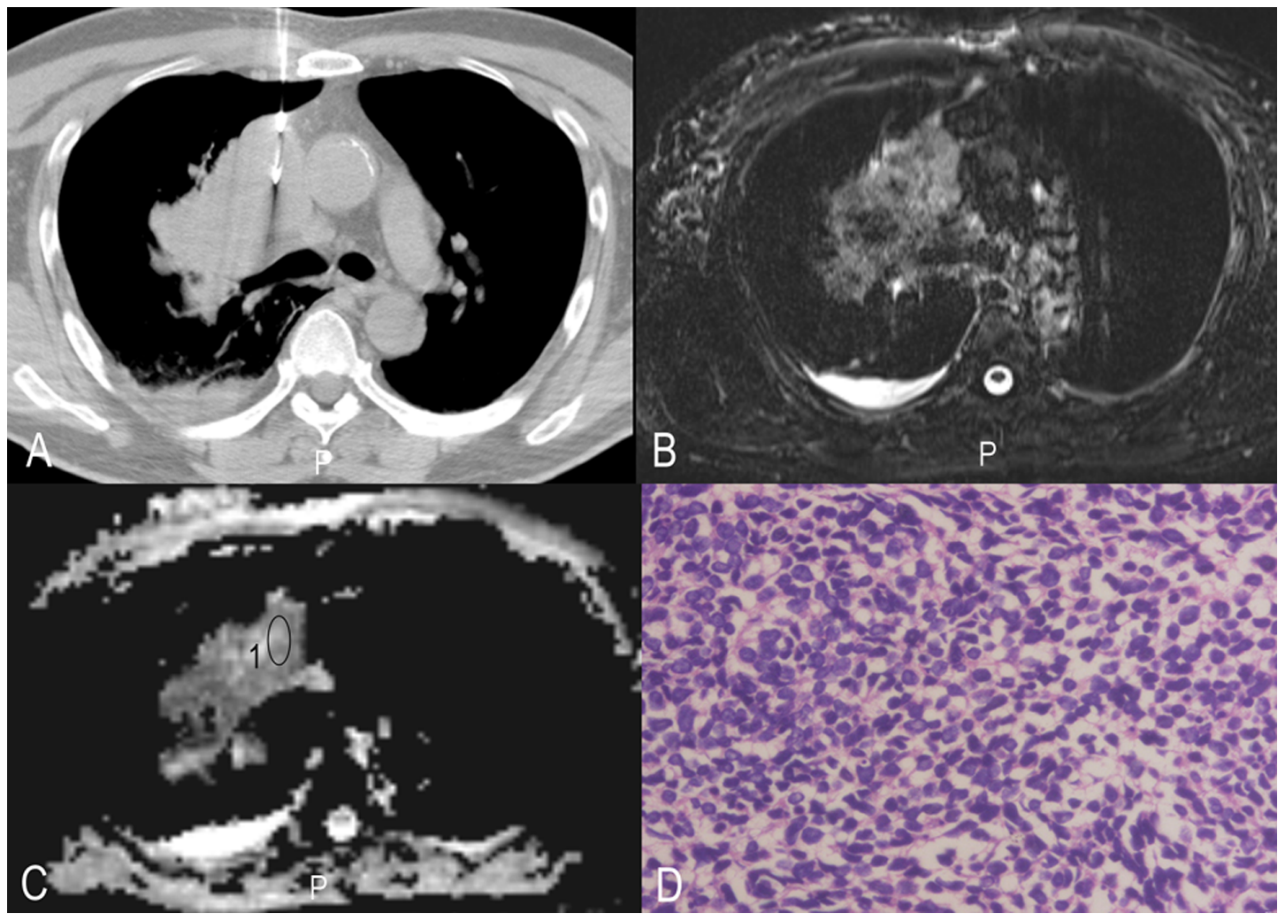
Histopathologic correlation of MR imaging data with biopsy specimens in a 48-year-old woman with small cell lung cancer. A, CT-guided biopsy; B, T2WI-TIRM; C, ADC map and ROI where ADCs have been measured are illustrated, 1 = ROI; D, corresponding histopathologic results for the tumour sample (original magnification, HE×400).

All MR imaging was performed on a 3.0 Tesla scanner (Trio Tim, Siemens, Erlangen, Germany) in combination with an 8 channel phase array coil. DWI was obtained during free breathing [14]. DWI sequences were performed with 5000/72 (repetition time msec/echo time msec), a section thickness of 4 mm, an intersection gap of 0.8 mm, and a field of view of 400×400 mm. Three signals were acquired per image with diffusion-sensitising gradients in three orthogonal planes and b values of 50, and 1000 sec/mm^2^ during free breathing. To minimise the influence of respiratory movement on data quality, two approaches were taken: (1) respiratory training, and (2) guided free breathing (instructions from the imaging radiographer). Fat was suppressed by placing a frequency-selective radiofrequency pulse before the pulse sequence. All of the ADCs (ADCmean, ADCmin) were measured by two chest radiologists with 8 and 11 years of experience. In outlining the ROI for measurement of the DWI metrics, we attempted to draw on the solid part of the tumours by imaging of the CT-guided biopsy (Siemens Plus 4), see Figure 1–3.

### Tumour Cellularity Analysis

Histopathologic specimens were coated with standard marking ink (Hematoxylin-Eosin Staining Kit, Beyotime, Jiangsu, China) and fixed in 10% buffered formaldehyde for 24 hours. The histopathology results were the standard of reference for the analysis of cellularity. All histopathologic specimens were reviewed by a histopathologist with 20 years of experience in histopathology.

Tumour cellularity was calculated from 5 arbitrarily selected high-power fields in each specimen using a computer program (ImageJ; National Institutes of Health, Bethesda, MD) and the following algorithm: first, digitised high-power (×40 objective) fields were taken from the original microscopic images with a 512×512 display matrix and 8-bit grey scale, which were obtained using a digital microscope camera (Olympus DP12; Olympus, Tokyo, Japan). Binary image data were then derived from the sample images using a threshold value estimated from histogram analysis of the sample images. Finally, the software calculated the cellularity based on the number of separate dots produced by the binary procedure. The automated counting system performed to within 5% tolerance in all specimens. The automated settings were identical for all specimens and cell counts. The results for the 5 high-power-field cell counts were used to derive the median cell count for the specimen. This method of measuring cellularity is similar to the methods used by other investigators who compared diffusion-weighted imaging features with tumour cellularity [15].

### Statistical Analysis

Statistical analyses were performed using statistical software (SPSS, version 18.0; SPSS Inc, Chicago, IL, USA). The Kolmogorov-Smirnov test was used to assess the deviation from normal distribution for all data. Intraclass correlation coefficients (ICC) were calculated to evaluate the inter-observer variance in the DWI metrics measurements. To compare the ADCmean values and ADCmin values among histologic types of adenocarcinoma, squamous cell carcinoma and small cell lung cancer, analysis of variance was used. The post hoc hypothesis testing was performed according to the Fisher protected least significant difference method. In addition, Pearson correlation coefficients were calculated to evaluate the correlation between ADCmean values/ADCmin values and the tumour cellularity of lung carcinomas. A P value of less than 0.05 was considered to indicate a statistically significant difference.

## Results

### ADC Values in Lung Cancer

All DWI images clearly showed that there were no significant susceptibility artefacts and that they were suitable for further evaluation. All ADCmean, ADCmin and tumour cellularity values were successfully obtained (Table 1, Figure 1–3). The ICCs between the two radiologists for the measurement of ADCmean, ADCmin and tumour cellularity values were 0.93, 0.92, and 0.88, respectively. The final values were the mean of the two measurements. The ADCmean value of the primary tumours was (1.00±0.15) × 10^−3^ mm^2^/s in total, (0.89±0.13) × 10^−3^ mm^2^/s in small cell lung cancer, (1.07±0.12) × 10^−3^ mm^2^/s in adenocarcinoma, and (0.88±0.10) × 10^−3^ mm^2^/s in squamous cell carcinoma. Each set of data was in line with or approximated a normal distribution (all P>0.1). The analysis of variance showed significant differences among three groups (P<0.05). The ADCmin value of the primary tumours was (0.80±0.15) × 10^−3^ mm^2^/s in total, (0.67±0.13) × 10^−3^ mm^2^/s in small cell lung cancer, (0.86±0.13) × 10^−3^ mm^2^/s in adenocarcinoma, and (0.73±0.13) × 10^−3^ mm^2^/s in squamous cell carcinoma. Each set of data was in line with or approximated a normal distribution (all P>0.1). The analysis of variance showed significant differences among three groups (P<0.05).

**Table 1:** ADCs and Cellularity among Histologic Types of Lung Cancer (Data is expressed as mean ± standard deviation).

Histologic Type	n	ADCmean (10^−3^ mm^2^/s)	ADCmin (10^−3^ mm^2^/s)	Cellularity (%)
Small cell lung cancer	9	0.89±0.13	0.67±0.13	18.3±3.5
Adenocarcinoma	37	1.07±0.12	0.86±0.14	14.9±2.6
Squamous cell carcinoma	14	0.88±0.10	0.73±0.12	20.6±4.4
All	60	1.00±0.15	0.80±0.15	16.7±4.0

### Tumour Cellularity in Lung Cancer

The NCR of the primary tumours was (16.7±4.0) % in total, (18.3±3.5) % in small cell lung cancer, (14.9±2.6) % in adenocarcinoma, and (20.6±4.4) % in squamous cell carcinoma. Each set of data was in line with or approximated a normal distribution (all P>0.1). The analysis of variance showed significant differences among three groups (P<0.05). Among these, the NCRs of squamous cell carcinoma ((20.6±4.4) %) and small cell lung cancer ((14.9±2.6) %) were higher than that of adenocarcinoma ((14.9±2.6) %), and the difference was statistically significant (P<0.01 and P = 0.002, respectively). The NCR of squamous cell carcinoma cells was greater than that of small cell lung cancer cells, but the difference was not statistically significant (P = 0.198).

### The Correlation between ADC Value and Cellularity in Lung Cancer

**Figure 4:**
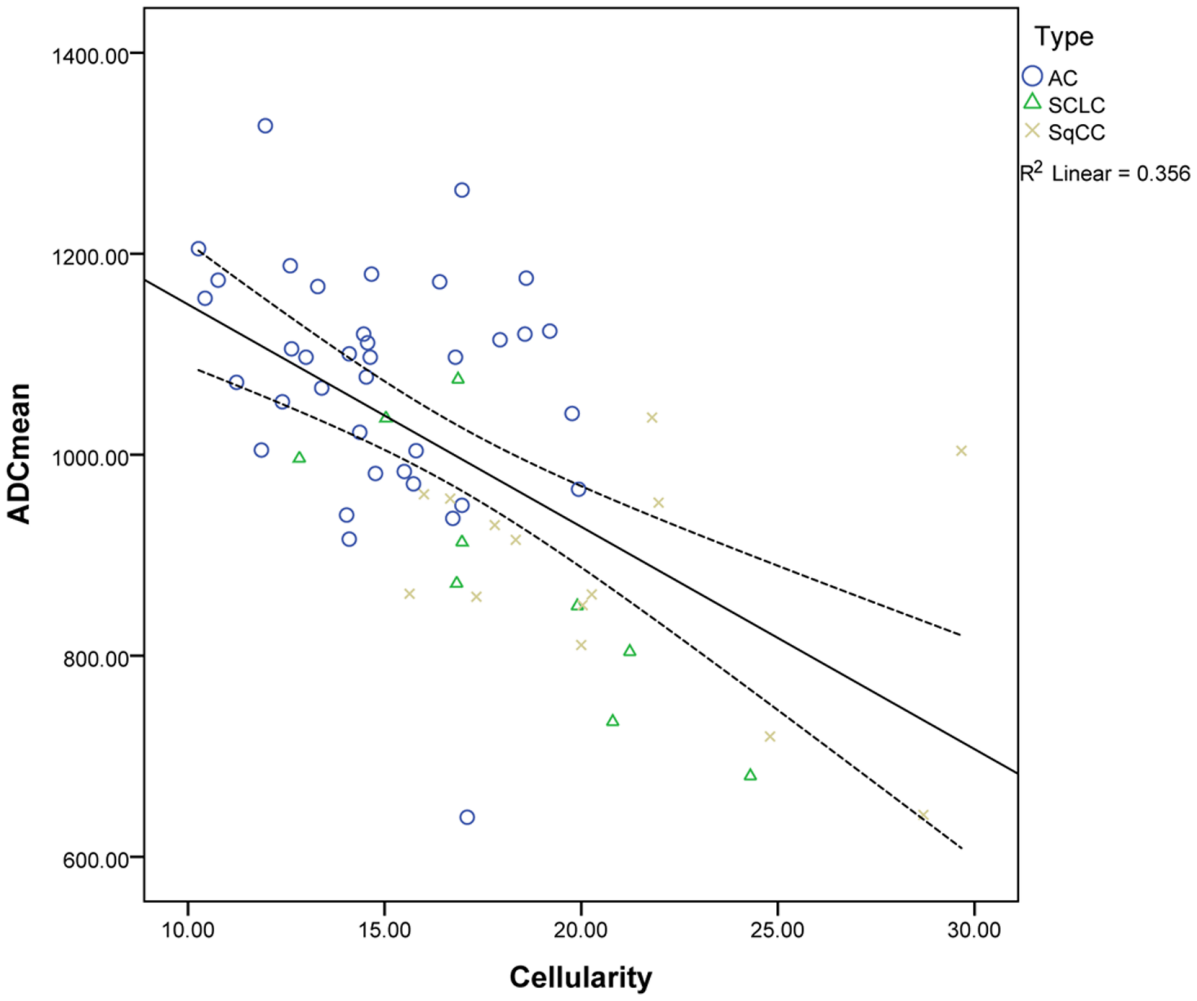
Graph shows the correlation between ADCmean and tumour cellularity of lung cancer. AC, adenocarcinoma; SqCC, squamous cell carcinoma; SLCC, small cell lung cancer.

**Figure 5:**
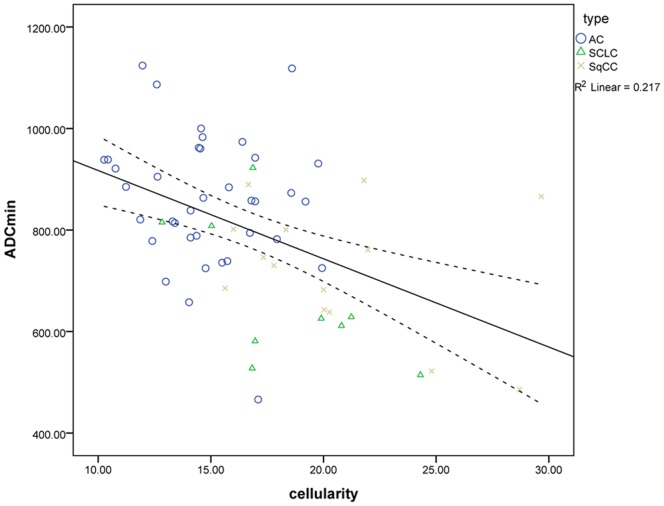
Graph shows the correlation between ADCmin and tumour cellularity of lung cancer. AC, adenocarcinoma; SqCC, squamous cell carcinoma; SLCC, small cell lung cancer.

Correlation analysis showed that the ADCmean value and lung tumour cellularity was significantly negatively correlated (r = −0.60, P<0.001), see Figure 4; Meanwhile, ADCmin values and lung tumour cellularity were significantly negatively correlated (r = −0.47, P<0.001), see Figure 5.

## Discussion

We have performed correlation analyses for both ADCmean and ADCmin with tumour cellularity and have shown that both ADCmean and ADCmin had a negative relationship with tumour cellularity in lung cancer. Matoba *et al* [16] performed correlation analysis between ADCmean and tumour cellularity in 9 cases of lung cancer and found the similar results as we did. This result implies that DWI might have great potential application in the diagnosis and treatment of lung cancer. Beyond distinguishing lung cancer from benign tumours and central lung cancer from its associated atelectasis, as well as lymph node staging of lung cancer, DWI might be used to evaluate therapeutic efficacy after early treatment. Morphological methods, such as the WHO standards and RECIST criteria, are the most frequently used methods to evaluate tumour therapy in the clinic. Compared to these methods, ADC values appear to change significantly much earlier than morphological changes occur [17], [18]. DWI is also useful for CT-guided percutaneous lung biopsy, which is the most common minimally invasive means to access histopathologic specimens. Due to the heterogeneity of lung tissue distribution, the positive biopsy rate is approximately 80% [19] to 94.8% [20], and the accuracy needs to be improved. The auxiliary DWI images and ADC map help to avoid non-neoplastic tumours and select ROIs with relatively higher density for puncture, which may improve the accuracy of lung biopsies.

Our results experimentally verifies the negative relationship between ADC and cellularity in a more quantitative manner and may be of value for more accurately modeling the diffusion process. Higher nuclear/cytoplasm ratio will reduce extracellular space which may lead to lower apparent diffusion coefficient [2]. In the other hand, higher cellularity will lead to more cell membranes that the molecules of water (or particles of any kind) would have to diffuse through. This addition of barriers may also lead to lower apparent diffusion coefficient. However, the regression model of ADC, which we have established, cannot explain the observed data perfectly (r = −0.60). In addition to cell density, the nuclear/cytoplasm ratio, and large nuclei, the diffusion of water molecules is also affected by many other characteristic features of tumours, including perfusion, the amount of intracellular macromolecular proteins, cell proliferation, and extracellular space relative to normal tissue [2], which may be one of the reasons why the model did not fit the data exactly.

Both ADCmin and ADCmean values have been reported to correlate inversely with glial and nonglial tumour cellularity as a result of the restricted diffusion of water [15], [21]–[23]. In the subgroup analysis of Chen *et al* [7], the ADCmean values were reported to correlate more inversely than the ADCmin values, and there were no significant differences between subgroups of ADCmean and ADCmin (r = −0.70 and −0.60). There were also no significant differences between these values (−0.60 and −0.47) in our study, which means that the measurement of the ADC value and tumour cellularity is consistent and stable.

Our results show that the tumour cellularity of different histological types of lung cancer is not the same. The results from the studies of Matoba *et al* [16] and Razek *et al* [24] also show that the cellularity of adenocarcinoma was significantly lower than that of squamous cell carcinoma in non-small cell lung cancer. Therefore, we speculate that the ADC values might provide some value to distinguish these two different histological types of lung cancer. However, further large, prospective studies are warranted to validate these findings.

The indicators to evaluate tumour cellularity included cell density, nuclear-cytoplasmic ratio and nuclear cell ratio. Cell density was calculated by dividing the total area of tumour cell nuclei by the area of the histology section. The nuclear-cytoplasmic ratio was calculated by dividing the percentage of the nuclear area by the percentage of the cytoplasmic area. The nuclear cell ratio was calculated by dividing the percentage of the nuclear area by the percentage of the cell area. Compared to cell density, the nuclear-cytoplasmic ratio and the nuclear cell ratio can not only reflect cell density but can also reflect the nuclear/cytoplasm ratio and large nuclei, as all of these factors affect the ADC values [2].

There are some limitations to this study. First, the study only included adenocarcinoma, squamous cell carcinoma and small cell lung cancer, and other rare histological types of lung cancer, such as large cell lung cancer, could not be included. Second, the total cell density was considered for comparison, so tumour cells were not separated from non-tumour cells. Physiologically, however, non-tumour cells also affect the water diffusivity within the tissue, and most previous reports did not distinguish tumour cells. Third, this study only focussed on the correlation of tumour cellularity and ADC value in the biopsy site, which cannot represent the whole tumour.

In conclusion, our study showed that the ADCmean value and the ADCmin value were significantly negatively correlated with tumour cellularity in patients with lung cancer. However, tumour cellularity most likely is not the sole determinant of the ADC.

## Supporting Information

Figure S1 Flowchart illustrating the selection of studies.
(TIF)
