# Supplementary figures and images for: Relationship between Apparent Diffusion Coefficient and Tumour Cellularity in Lung Cancer
Source: PLoS One. 2014 Jun 11;9(6):e99865. doi: 10.1371/journal.pone.0099865 (PMC4053522; doi:10.1371/journal.pone.0099865)

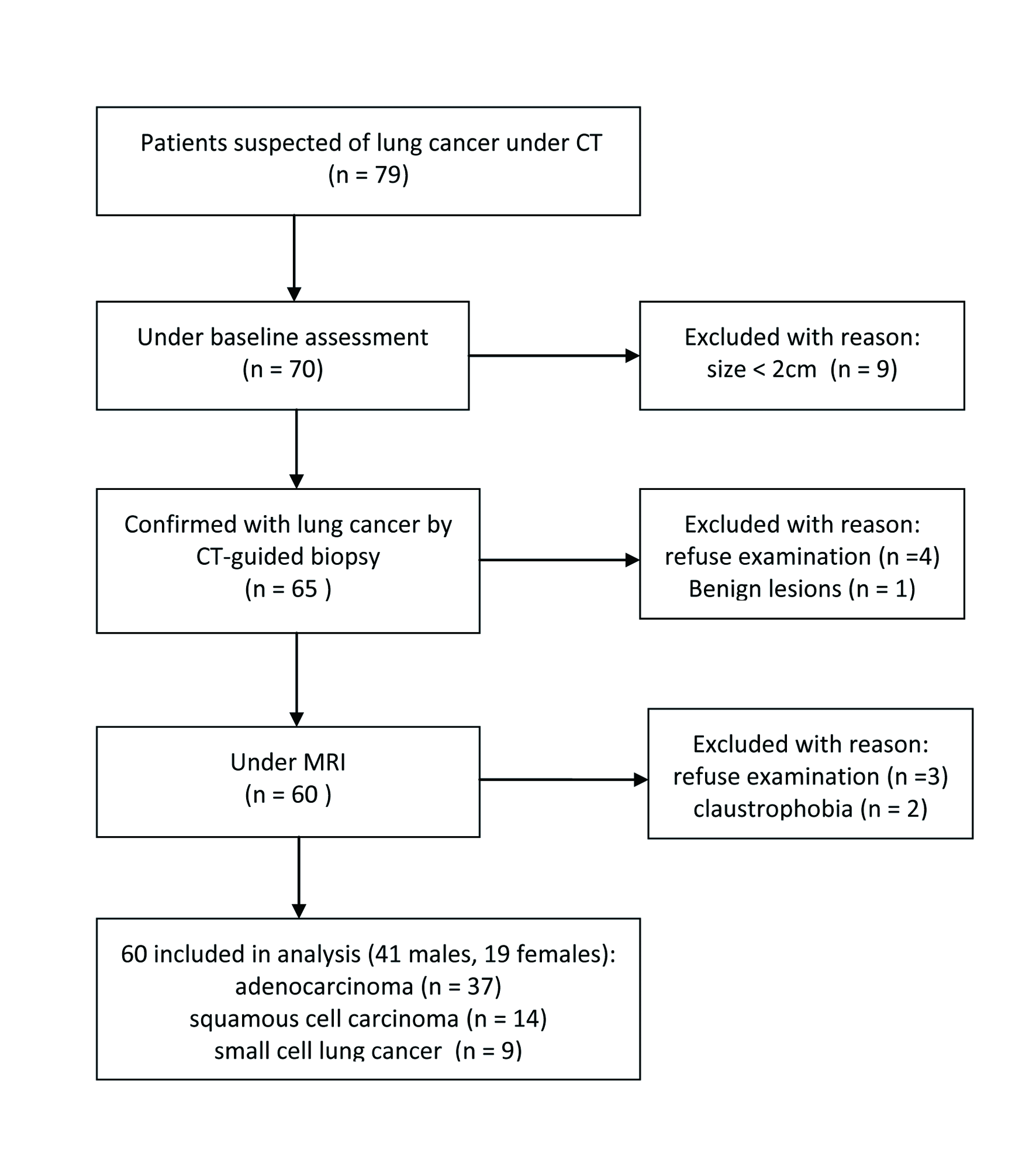

Supplement: Figure S1 — Flowchart illustrating the selection of studies. (TIF) [file pone.0099865.s001.tif]
